# Long‐term functional prognosis and related factors of spinal cord stimulation in patients with disorders of consciousness

**DOI:** 10.1111/cns.13870

**Published:** 2022-05-20

**Authors:** Yi Yang, Qiheng He, Xiaoyu Xia, Yuanyuan Dang, Xueling Chen, Jianghong He, Jizong Zhao

**Affiliations:** ^1^ Department of Neurosurgery, Beijing Tiantan Hospital Capital Medical University Beijing China; ^2^ Chinese Institute for Brain Research Beijing China; ^3^ Beijing Institute of Brain Disorders Beijing China; ^4^ China National Clinical Research Center for Neurological Diseases Beijing China; ^5^ Department of Neurosurgery Seventh Medical Center of PLA General Hospital Beijing China

**Keywords:** disorders of consciousness, minimally conscious state, prognosis, related factors, spinal cord stimulation, vegetative state/unresponsive wakefulness syndrome

## Abstract

**Introduction:**

The treatment of patients with disorders of consciousness (DoC) remains a challenging issue, and spinal cord stimulation (SCS) has been reported to be a promising treatment for DoC in some studies.

**Aims:**

This study explores the efficiency of SCS in treating patients with DoC at different consciousness levels, including the vegetative state/unresponsive wakefulness syndrome (VS/UWS) and the minimally conscious state (MCS) and summarizes and analyzes the long‐term effect and related factors of SCS in patients with DoC.

**Results:**

An overall positive outcome was reached in 35 of 110 patients (31.8%). Among patients with positive outcomes, the MCS group improved 45.53% more than VS/UWS group, and this difference was statistically significant. In terms of the recommendation standard, positive outcomes occurred in 33 patients (94.3%) in the highly recommended group and 2 patients (5.7%) in the weakly recommended group (*p* < 0.001). After adjustment for potential covariables, young age (age ≤ 19 years old) (*p* = 0.045) and MCS (*p* < 0.001) were significantly correlated with positive outcome. A nomogram based on age, state of consciousness, and pathogeny showed good predictive performance, with a c‐index of 0.794. The Hosmer–Lemeshow goodness‐of‐fit test showed that the model was well calibrated (*χ* ^2^ = 3.846, *p* = 0.871).

**Conclusions:**

SCS is one of the most feasible treatments for patients with DoC, especially for patients with MCS. Younger age is significantly associated with better outcomes and could therefore serve as a basis for preoperative screening. However, more evidence‐based randomized controlled trials are needed to confirm the efficacy of the treatment.

## Graphical abstract

With developments in neurocritical care, the number of patients with disorders of consciousness (DoC) is rapidly growing but the treatment remains challenging. The paper explores the efficiency of SCS in treating patients with DoC at different consciousness levels and summarizes the long‐term functional prognosis and related factors of SCS in patients with DoC.

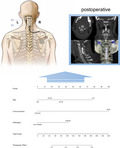

## 1 INTRODUCTION

With developments in neurocritical care, the number of patients with disorders of consciousness (DoC) is rapidly growing as more patients with severe brain injury survive.^1^, ^2^ The concept of DoC encompasses a wide spectrum of diseases depending on wakefulness and awareness, including coma, the vegetative state/unresponsive wakefulness syndrome (VS/UWS), and the minimally conscious state (MCS). The overall possibility for spontaneous recovery of consciousness from VS/UWS is very low, and the possibility of recovery decreases as the duration of VS/UWS increases.^3^ As patients in MCS show inconsistent but discernible signs of consciousness, such as command‐following or other purposeful behaviors, their probability of recovery is higher.^4^ The management of DoC is a challenge in the fields of medicine and neuroscience research because of the lack of evidence‐based treatments.^5^, ^6^ Clinicians have tried treatments including pharmacological agents, hyperbaric oxygen therapy, and sensory and environmental stimulation therapy, but none of them achieved good results. Thus, promising methods to enhance consciousness are still needed.

Spinal cord stimulation (SCS) is a therapeutic technique that owes its inception to the concept of gate control theory, proposed by Melzack in 1965; the first reported dorsal column stimulation came 2 years later.^7^, ^8^ Since then, SCS has been classically used for pain and spasticity management.^9^, ^10^, ^11^, ^12^ According to the literature, Kanno et al. were the first to apply this technique in patients with DoC, and their work showed encouraging results. In the treatment of DoC, electrodes were implanted along the midline of the posterior epidural space of the C2‐C4 level and delivered electric stimulation to the circuitry governing awareness.^13^ However, since 1988, only 10 papers involving 308 VS patients have been published; 51.6% of these patients showed improvements in their clinical status and environmental interactions.^14^, ^15^ The existing data are thus very limited, and the related factors that may influence the long‐term effect are unclear. In this study, we retrospectively analyzed the medical records of 110 patients with chronic DoC treated by SCS, summarized the results of follow‐up, analyzed the overall effectiveness rate and the related factors influencing the long‐term effect, and discussed the potential factors that could serve as a basis for preoperative screening.

## 2 MATERIALS AND METHODS

### 2.1 Study participants

The data that support the findings of this study are available from the corresponding author upon reasonable request. We retrospectively consecutively recruited 121 patients with DoC who were treated with SCS at the Department of Disorders of Consciousness, PLA Army General Hospital, and the Department of Disorders of Consciousness, Beijing Tiantan Hospital, Capital Medical University, from September 1, 2011, to December 31, 2017 (Table S1). Because these patients could not understand and legally consent to our method of treatment, we explained to each patient's legal representative and/or close relative/s the options for treatment, the possible risks and benefits of our mode of treatment, and the nature of SCS for DoC. Once the realistic expectations had been explained, these legal representatives and/or family members were offered an informed consent document compatible with the legal and ethical committee regulations adopted at our institution. These regulations conform to the internationally adopted ethical standards for the performance of clinical treatment and research (the Declaration of Helsinki). Patients were enrolled in the study if their caregivers provided written informed consent to their participation. The study was approved by the ethics committee of PLA Army General Hospital (2011–0415) and ethics committee of Beijing Tiantan Hospital, Capital Medical University (2017–361‐01).

All cases of DoC in this study had a pathogeny of traumatic brain injury (TBI), stroke, or global anoxia. All patients met the criteria for DoC, and the duration of DoC was at least three months, as adopted from the recommendations of the Multi‐Society Task Force on the Persistent Vegetative State (1994).^3^, ^16^

### 2.2 Preoperative evaluation

#### 2.2.1 JFK coma recovery scale–revised (CRS‐R)

The consciousness state of each respondent was assessed using the CRS‐R.^17^ The CRS‐R consists of six subitems to evaluate the degrees of hearing, vision, movement, speech, communication, and arousal. All patients underwent repeated CRS‐R scoring (at least 5 times within 2 weeks) at the consciousness stage after their condition was stable (no complications such as fever or seizures); the scale was applied by trained professional raters, who took the highest score to assess the baseline consciousness level of the patients. CRS‐R scores were regularly reviewed after surgery and before discharge (once a week). After discharge, we scored the patients according to telephone or video call follow‐up data, and all scoring data were stored and compiled.

#### 2.2.2 Magnetic resonance imaging (MRI)

For eligible patients (no large skull defects, no skull repair, and no implanted shunt pumps), a GE HD750 3.0 T superconducting MR scanner (US, GE) was used to acquire normal and resting‐state functional MRI (fMRI) scans for the preoperative evaluation. We evaluated brain atrophy, assessed damage in key brain regions, and calculated the activation and connectivity of key brain networks as described in our previously published studies.^18^

#### 2.2.3 Electroencephalography (EEG)

EEG examination was performed on eligible patients at the preoperative evaluation stage. Data from regular EEG and concurrent transcranial magnetic stimulation and EEG were collected with MRI‐compatible EEG equipment (BrainAmp 64 MRplus from BrainProducts Company). The EEG cap included 64 leads and was placed according to the international standard 10–20 scale. The resting EEG data were recorded for at least 20 min.^19^ The clinical assessment of the EEG data was performed by experienced doctors, and further quantitative EEG analysis was performed to consider aspects such as power spectrum and sorting entropy.

#### 2.2.4 Mismatch negativity wave (MMN)

Some patients underwent MMN examination. A Guangzhou Runjie Medical Event‐Related Potentiometer was used along with four electrodes, located at F3, Fz, Cz, and F4. The bilateral earlobes were used as a reference, and the impedance was below 10 kΩ. The stimulus sounds were pure tones; the standard sound was 800 Hz (occurring in 90% of trials), and the deviant sound was 1000 Hz (occurring in 10% of trials). Each stimulus rose over the course of 5 ms each to an intensity of 75 dB, and the stimuli were presented at intervals of 1000 ms. During the analysis, the data segments exceeding 100 μV were removed for superposition and averaging, and the MMN amplitudes between 100 ms and 300 ms were calculated.

### 2.3 Recommended surgical criteria

In addition to subjective assessment criteria such as the CRS‐R scale, objective criteria such as MRI, EEG, and MMN are also used to identify hidden residual consciousness due to factors such as hemiplegia or aphasia.^20^, ^21^, ^22^, ^23^ Doctors comprehensively assess the patient's condition based on the above subjective and objective examination results, as reported by Giacino et al..^24^ The main criteria were 1. Focal brain damage <30%; 2. Probability of emerge >30% by fMRI and analyzed by pDOC R package^18^; 3. MMN >1^25^; 4. Synek grade less than III.^26^

### 2.4 Implantation of the stimulator

**FIGURE 1:**
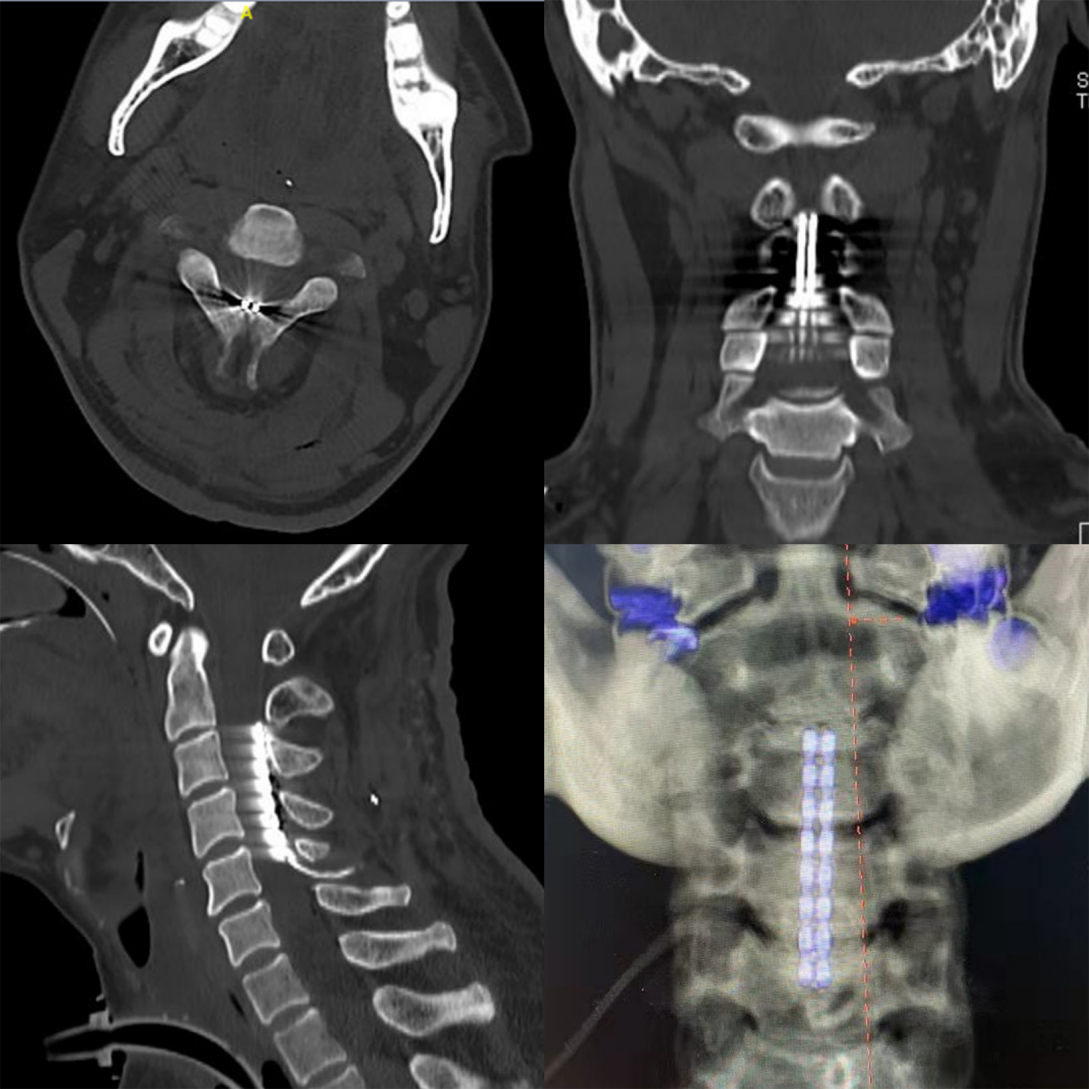
Cervical CT scan and reconstruction after SCS implantation. The electrode was implanted in the cervical spinal canal. Images 1–3 represent the axial, coronal, and sagittal views of cervical CT. Image 4 represents the sagittal view of VRT reconstruction

The C5 vertebra was positioned under a fluoroscope, and a midline incision measuring 5–7 cm was made under general anesthesia. After this incision was made, the C5 spinous process was exposed, and a 2 cm‐wide opening was made in the C5 spinous process and lamina. Using the expander included with the implantable electrode kit, the dura was loosened from the bone along with the midline at the C2‐4 levels, stopping at the upper limit of the opening in C5. After the marking scale showed that the expander had reached the spinal canal, a silicone model of the electrode was inserted into the C2‐C4 level. If the placement was correct, then the silicone model was replaced with a Medtronic 39,286 electrode. A temporary stimulator was used to test for adverse reactions to different frequencies and intensities, including abnormal heart rate, blood pressure, and muscle contractions in the limbs. Finally, 2 fixed anchors were inserted, and the outer segment of the electrode was fixed to the spinous process and paraspinal muscles of the C6 level to prevent the electrode from being pulled out. X‐ray images of an implanted electrode are shown in Figure 1.

### 2.5 Stimulation protocol

Stimulation was usually initiated 3–7 days after surgery. The stimulation protocol was applied during the daytime, approximately 12 hours per day. The cranial pair of electrodes was the negative pole, and the second pair of electrodes was the positive pole. The posterior columns were stimulated at a frequency of 70 Hz and a pulse width of 210 μs. The stimulation parameters were chosen to remain below the motor threshold, as a motor response usually occurs above 2 ~ 2.5 V. The current was applied in a repeating cycle of 15 min on and 15 min off.

### 2.6 Postoperative evaluation

All patients were followed up by their neurosurgeons, nursing staff, and relatives independently. The follow‐up was performed at three time points: 1 month, 6 months, and 12 months after the operation. The follow‐up included hospitalization records, outpatient review, and doctors' after‐the‐fact evaluations conducted face to face or through video. According to the GOS, the prognosis was judged first, and then the prognosis was classified based on the clinical symptoms measured by the CRS‐R scale. EEG examination was performed in hospitalized patients. Postoperative evaluations were performed according to the same criteria as the preoperative evaluations. Three groups of treated patients were classified according to results: excellent, effective, and unchanged. All patients served as their own controls. No other treatments and drugs that would modify cortical excitability were administered.

### 2.7 Statistical analysis

The preoperative CRS‐R score and the last follow‐up CRS‐R score were compared using a paired‐sample t‐test if the differences between them were normally distributed. Otherwise, the Wilcoxon signed‐rank test was used. K‐S test was used to judge the normality of continuous variables. The continuous variables of normal and skewness distribution were reported as mean (standard deviation, SD) and median (interquartile range, IQR), respectively, and the categorical variables were expressed as number (percentage). The results of MRI, EEG, and MMN were compared between the responsive group and the unresponsive group using Fisher's exact test. Outcomes were compared between the MCS group and the VS/UWS group via the chi‐square test or Fisher's exact test. The subgroup analysis was conducted by adding interaction effects of treatment, gender, duration, and pathogeny into the mixed effect model. The 95% confidence intervals were calculated in all analyses. All statistical analyses were performed using SAS version 9.4 (SAS Institute), and the significance threshold was a two‐sided *p* value of less than 0.05.

## 3 RESULTS

### 3.1 Baseline characteristics of patients with MCS and VS/UWS

From September 1, 2011, to December 31, 2017, SCS was performed in 110 patients, including 72 males (65.1%) and 38 females (34.9%). There were 13 (41.9%) female participants in the MCS group and 25 (31.6%) female participants in the VS/UWS group. The sex distribution did not differ between the MCS group and the VS/UWS group as determined by the chi‐square test (*p* = 0.178). The average age was 41.1 ± 13.8 years, with a maximum age of 71 years and a minimum age of 10 years. Seventy‐nine cases (71.8%) were diagnosed with VS/UWS, and 31 cases (28.2%) were diagnosed with MCS. The average duration of disease was 9.6 ± 12.6 months, with the shortest period being 3 months and the longest period being 84 months. There were 42 cases of trauma (38.2%), 33 cases of anoxia (30.0%), and 35 cases of stroke (31.8%) included in the study. There were no significant differences in age or pathogeny between the MCS and VS/UWS groups (*p* > 0.05). However, regarding the recommendation criteria, all MCS patients were in the highly recommended category, compared with only 25 of 79 (31.6%) VS/UWS patients (*p* < 0.001). The demographics of the patients are presented in Table 1.

**TABLE 1:** Baseline characteristics of the participants

Variables (%)	All patients (*n* = 110)	State of consciousness		*p* value
		MCS *(*n = 31)	VS/UWS (*n* = 79)	
Sex				0.307
Male	72(65.4)	18(58.1)	54(68.4)	
Female	38(34.5)	13(41.9)	25(31.6)	
Age (years)				0.108
≤19	11(10.0)	4(12.9)	7(8.9)	
20–39	35(31.8)	14(45.2)	21(26.6)	
40–60	56(50.9)	10(32.3)	46(58.2)	
>60	8(7.3)	3(9.7)	5(6.3)	
Pathogeny				0.510
Anoxia	33(30.0)	7(22.6)	26(32.9)	
Stroke	35(31.8)	10(32.3)	25(31.6)	
Trauma	42(38.2)	14(45.2)	28(35.4)	
Duration (months)				0.446
3–5	55(50.0)	14(45.2)	41(51.9)	
6–11	33(30.0)	12(38.7)	21(26.6)	
≥12	22(20.0)	5(16.1)	17(21.5)	
Recommendation criteria				
Highly recommended	56(50.9)	31(100)	25(31.6)	0.000
Weakly recommended	54(49.1)	0(0)	54(68.4)	

*p < 0.05, significant difference.

### 3.2 Clinical characteristics of patients according to therapeutic effect

As patients with DoC are often prone to complications, their general condition and consciousness may fluctuate or even decline over the 12 months after surgery. This study focuses on the improvement of consciousness in response to SCS surgery. Therefore, the criteria for judging the efficacy are as follows: 1. Those who continued to improve were judged according to the 12 months follow‐up results; 2. For patients who worsened due to complications, the data from the best follow‐up point were included. The overall effectiveness rate was 31.8%, and the results are shown in Table 2.

**TABLE 2:** Univariate analysis of related factors influencing prognosis

Variables (%)	All patients (*n* = 110)	Therapeutic effect			*χ* ^2^	*p* value
		Positive (*n* = 35)	Unchanged (*n* = 68)	Dead (*n* = 7)		
Sex					3.456	0.178
Male	72(65.4)	19(54.3)	49(72.1)	4(57.1)		
Female	38(34.5)	16(45.7)	19(27.9)	3(42.9)		
Age (years)					16.944	0.009
≤19	11(10.0)	8(22.9)	3(4.4)	1(14.3)		
20–39	35(31.8)	12(34.3)	19(27.9)	1(14.3)		
40–60	56(50.9)	13(37.1)	42(61.8)	3(42.9)		
>60	8(7.3)	2(5.7)	4(5.9)	2(28.6)		
State of consciousness					22.093	0.000
MCS	31(28.2)	20(57.1)	11(16.2)	0(0)		
VS/UWS	79(71.8)	15(42.9)	57(83.8)	7(100)		
Pathogeny					14.443	0.006
Anoxia	33(30.0)	9(25.7)	23(33.8)	1(14.3)		
Stroke	35(31.8)	7(20.0)	22(32.4)	6(85.7)		
Trauma	42(38.2)	19(54.3)	23(33.8)	0(0)		
Duration (months)					5.860	0.210
3–5	55(50.0)	14(40.0)	37(54.4)	4(57.1)		
6–11	33(30.0)	10(28.6)	22(32.4)	1(14.3)		
≥12	22(20.0)	11(31.4)	9(13.2)	2(28.6)		
Recommendation criteria					43.643	0.000
Highly recommended	56(50.9)	33(94.3)	22(32.3)	1(14.3)		
Weakly recommended	54(49.1)	2(5.7)	46(67.6)	6(85.7)		

*p < 0.05, significant difference.

Overall, 20 of 31 (64.52%) patients in the MCS group had positive outcomes, while only 15 of 79 (18.99%) in the VS/UWS group had positive outcomes. The MCS group showed 45.53% more improvement than VS/UWS group, and chi‐square (*χ* ^2^) testing confirmed that this difference was (*p* < 0.001). Regarding the recommendation standard, 33 of 56 (58.9%) patients classified in the “highly recommended” group achieved positive outcomes, while 2 of 54 in the “weaker recommendation” group achieved positive outcomes; these groups accounted for 94.3% and 5.7% of all positive outcomes, respectively. The “highly recommended” group had a significantly better rate of positive outcomes than the “weakly recommended” group (*χ* ^2^ = 43.643; *p* < 0.001).

Among the 72 male patients enrolled in the study, the procedure was effective in 26.4% (19/72). Among the 38 female patients, the effectiveness rate was 42.1% (16/38). No significant difference was found between sexes (*χ* ^2^ = 3.456; *p* = 0.178). By age group, the positive outcome rates were 72.7% (8/11) in patients aged ≤19 years, 34.3% (12/35) in patients aged 20 ~ 39 years, 23.2% (13/56) in patients aged 40 ~ 60 years, and 25.0% (2/8) in patients aged >60 years; these rates were significantly different (*χ* ^2^ = 16.944; *p* = 0.009).

Regarding pathogeny, positive outcomes were achieved in nineteen of 42 (45.2%) patients in the trauma group, nine of 33 (27.3%) in the anoxia group, and seven of 35 (20.0%) in the stroke group (*χ* ^2^ = 14.443; *p* = 0.006). Regarding the duration of DoC, fourteen of 55 (25.5%) patients with a disease duration of 3–5 months achieved positive outcomes, compared with ten of 33 (30.3%) with a duration of 6–11 months, and eleven of 22 with a duration of ≥12 months. There was no significant difference between groups (*p* = 0.210). The average duration was 12.47 ± 11 months in the positive outcome group and 10.19 ± 13.87 months in the unchanged group.

### 3.3 Analysis of factors associated with therapeutic effects

We analyzed the potential factors associated with therapeutic effects in patients with DoC. The results are shown in Table 3. Sex, age, state of consciousness, pathogeny, and duration were all considered in the mixed model. However, after adjusting for all potential covariables, the results showed that age ≤ 19 (*p* = 0.045) and MCS (*p* < 0.001) were related factors associated with positive outcomes.

**TABLE 3:** Multivariate analysis of related factors influencing prognosis

Variables	Degrees of freedom	Estimation	Standard error	*χ* ^2^	*p* value
Intercept	1	0.1224	0.349	0.123	0.726
Sex					
Female	1	−0.2184	0.2719	0.645	0.422
Male					*
Age (years)					
≤19	1	−1.3176	0.6566	4.027	0.045
20–39	1	0.4777	0.4588	1.084	0.298
40–60	1	0.1704	0.4437	0.148	0.701
>60					*
State of consciousness					
MCS	1	−1.2212	0.2885	17.920	0.000
VS/UWS					*
Pathogeny					
Anoxia	1	−0.0813	0.3754	0.047	0.828
Stroke	1	0.5407	0.3904	1.919	0.166
Trauma					*
Duration (months)					
3–5	1	0.3271	0.3506	0.871	0.351
6–11	1	0.3261	0.3776	0.746	0.388
≥12					*

*p < 0.05, significant difference.

### 3.4 Nomogram of consciousness functional prognosis

**FIGURE 2:**
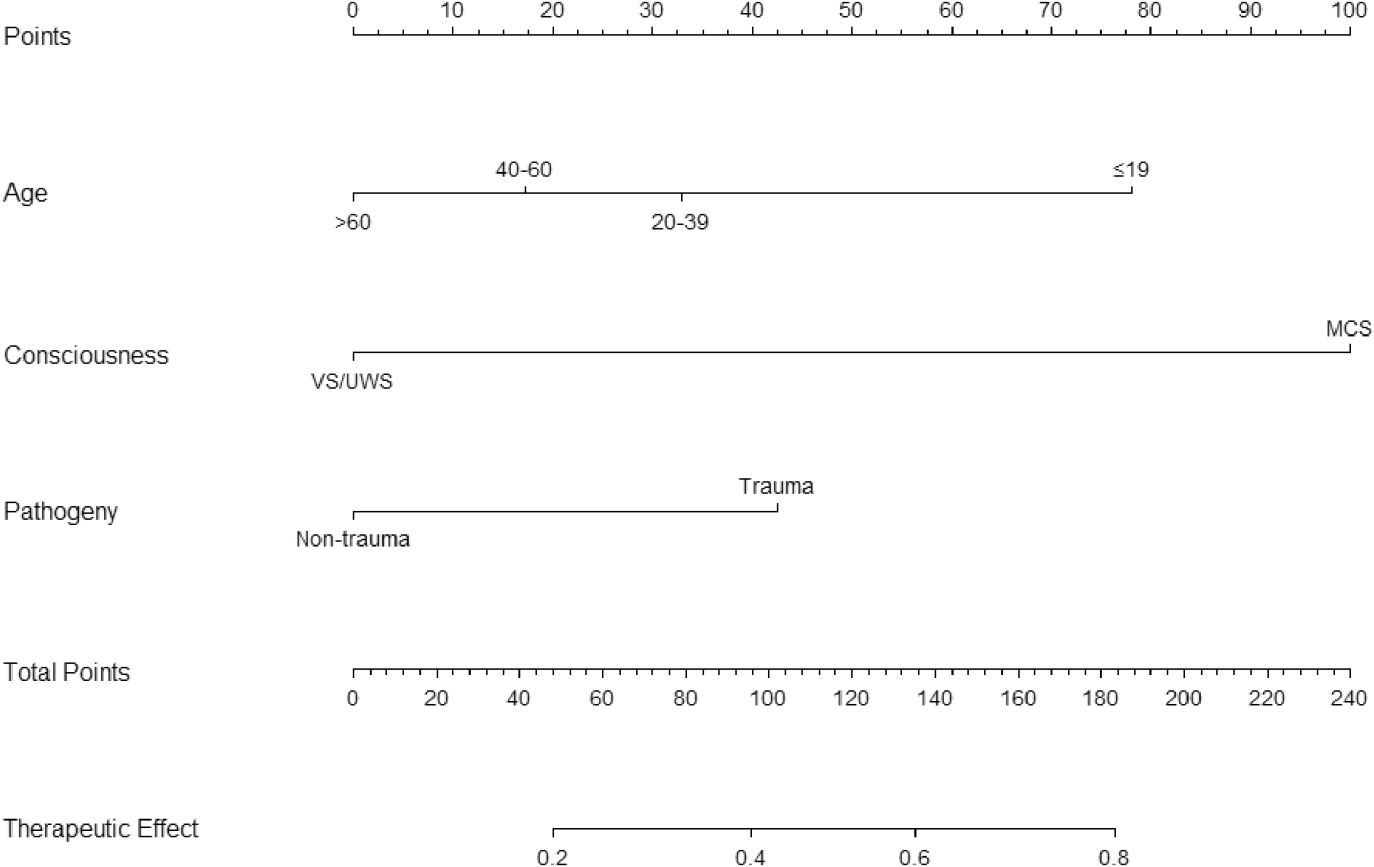
Nomogram of functional prognosis in patients with DOC receiving SCS treatment. Pathogeny is classified as trauma or non‐trauma (including stroke and anoxia). Therapeutic effect represents the possibility of effective possibility of SCS treatment in patients with DOC, which ranges from 0 (unchanged or death) to 1 (effective)

We constructed a nomogram based on potential related factors including age, state of consciousness, and pathogeny. The nomogram achieved a c‐index of 0.794, which reflects good predictive performance. The nomogram is shown in Figure 2. We also generated calibration curves for the nomogram, which are shown in Figure S1. The mean absolute error reached 0.03. Then, we performed the Hosmer–Lemeshow goodness‐of‐fit test, which showed that the model was well calibrated (χ^2^ = 3.846, *p* = 0.871).

### 3.5 Complications

During the early postoperative period (<1 month), 4 patients experienced subcutaneous hematoma or effusion in the chest wall, and 3 patients experienced incision dehiscence or poor wound healing. All cases were treated with early drainage, compression or debridement and suturing, and no subsequent adverse events occurred.

During the initial stimulation period, 1 case of frequent vomiting and 3 cases of epilepsy‐like actions were reported. The adverse reactions were alleviated after the parameters were adjusted and the stimulation intensity was reduced. In one case, the implant was removed due to skin rupture.

## 4 DISCUSSION

To date, clinical evidence of the treatment of chronic DoC by SCS is still limited. In the early 1980s, Komai first reported SCS treatment for patients in the persistent VS (PVS); in that study, 56 (43%) of 130 cases recovered. However, there was no concept of MCS at that time; therefore, some of the cases may have been misdiagnosed. In 2012, Yamamoto performed SCS procedures on 10 patients in MCS, and 7 (70%) of those patients showed significant improvements in consciousness.^27^ In 2013, Yamamoto reported another 10 MCS patients who underwent SCS operation; 5 (50%) of those patients recovered consciousness.^28^ At present, SCS is believed to be effective in some cases, with a total effectiveness rate of 20%–40%. We also reviewed the literature and found that 318 SCS patients were treated with SCS from 1988 to 2012. Overall, a clinical response was reported in 166 patients (52.2%), with improved neurological function and arousal.

Studies have shown that these positive effects on the prognosis of patients with DoC are probably mediated by stimulation of the reticulo‐thalamo‐cortical pathway and by an increase in cerebral blood flow induced by SCS. In 1985, Hosobuchi found that cervical SCS was able to increase cerebral blood flow (CBF) as measured by ^133^Xe imaging.^29^ There is also increasing interest in the ability of SCS to promote cortical neuroplasticity and improve functional activity in the brain circuits that are the neuronal substrates of consciousness and coma.^30^, ^31^, ^32^, ^33^, ^34^ For instance, Yamamoto et al. reported that SCS increased CBF diffusely throughout the brain in MCS patients, with an increase in more than 22% during the stimulation period compared with the period before stimulation.^28^

In light of these mechanistic and clinical studies, related factors affecting the outcomes of SCS treatment are being discussed. To date, clinical SCS experience in patients with DoC is limited to uncontrolled, small‐sample observational studies. Among the limited studies, Yamamoto et al. performed an observational series of 10 patients with DoC receiving SCS at 5 Hz and found that 8 had regained full consciousness by the 1‐year follow‐up.^28^ However, parameters including amplitude, frequency, pulse width, and duration were different across studies, which limits their comparability. Our previous studies measured the effects of SCS on EEG in MCS patients and the frequency‐specific effects on neurophysiological activity, finding that 70 Hz is the most effective frequency for the treatment of DoC.^35^

Thus, we carried out 110 SCS operations for DoC using a consistent, optimized set of parameters. Through a summary of previous medical records, continuous follow‐up, and statistical analysis of data, we found that the overall effectiveness rate of SCS for the improvement of DoC was 31.8%. Patients were further classified into the VS/UWS group and the MCS group according to their CRS‐R scores. In our study, the effectiveness rate in the MCS group was 64.5%, which was significantly higher than that in the VS/UWS group (19.0%). Reversible DoC can be the result of multiple mechanisms that globally alter neuronal function‐ or disable‐specific circuits.^36^ Patients with MCS can follow certain commands, even if their responses can be detected only as specific components of brain signals.^37^, ^38^ Therefore, global connectivity and vital circuits still exist in MCS patients, providing a basis for subsequent functional recovery through SCS. This may explain the superior outcome in patients with MCS compared with VS/UWS. We also found that the curative effect gradually decreased with age and had no relationship with sex. Research on brain development has shown high neuroplasticity throughout adolescence and young adulthood.^39^ Meanwhile, metabolic activity is also highest in young brains; such factors explain why younger patients were more likely to achieve positive outcomes.^40^, ^41^ Patients whose condition originated from trauma (45.2%) had significantly better outcomes than those with etiologies other than trauma (23.5%). This may be because most trauma patients have local deficits, whereas anoxia causes diffuse brain damage, which is more likely to affect the connectivity of the entire brain and thus impede recovery from DoC. Meanwhile, we found that a longer duration of DoC did not predict a worse outcome of SCS. It is possible that the timely stimulation of the remaining circuits prevented brain function from degrading in our study. Traditionally, etiology, duration of disease, and age are considered important factors affecting outcomes. However, according to our long‐term clinical observation and adjusted analysis, age, and severity of DoC are the most important factors that influence the long‐term prognosis. To further explore the impact of these factors on the therapeutic effect, we visualized potential‐related factors using a nomogram. The result of the Hosmer–Lemeshow goodness‐of‐fit test also showed that the model was well calibrated. The results demonstrate the good performance of these factors in predicting therapeutic effects. Therefore, it is vital to evaluate patients' consciousness level and consider age as a preoperative screening index because these variables have the most important influence on prognosis. The duration of disease is not always consistent with the consciousness level but rather influences the complications caused by prolonged DoC. This suggests that even for patients with a long duration of DoC, treatment is advisable, and such patients are still able to emerge from DoC.

Meanwhile, we summarized and verified the recommendation standards through clinical exploration. According to these standards, 56 patients were classified in the “highly recommended” group, and treatment was effective in 33 of those patients. The results also showed that the prognosis in the “highly recommended” group (58.9%) was significantly better than that in the “weaker recommendation” group (3.7%), emphasizing the important role of the comprehensive application of clinical and auxiliary technologies in preoperative evaluation. MCS patients were found to retain more brain function and have a better prognosis than VS/UWS patients. However, the CRS‐R, a behavioral evaluation method, has a misdiagnosis rate of 43%, which may easily lead to the overlooking of patients who have a chance of emerging. In our study, 15 patients (approximately 20%) in the VS group achieved positive outcomes. Neuroimaging and neuroelectrophysiological indicators are incorporated into the recommended surgical standards. These standards identified more positive cases than CRS‐R score screening; 11 of 15 patients in the VS group who achieved positive outcomes were included in the “highly recommended” group. The findings not only suggest that the operation recommendation standard is of high clinical practicability but also alert us to be more cautious about operating on the “weakly recommended” group in the future, even if their family members are strongly in favor of it.

Complications such as subcutaneous hematoma, effusion, incision dehiscence, and poor wound healing may occur in the early postoperative period (within 1 month) and are related not only to surgical factors but also to malnutrition and other factors associated with patients' general condition. Very few serious adverse reactions occurred during the initial stimulation. However, through clinical observation, we found that as the stimulation intensity increased (mainly the voltage), patients gradually began to show discomfort, such as pained expressions, limb flexion, or rigidity. The program should be adjusted to an appropriate stimulation intensity to avoid inducing excessive discomfort. The patients' tolerance to stimulation increases with the prolongation of treatment period, but when the consciousness level markedly increases, the patients' tolerance to stimulation often decreases rapidly. The discomfort feedback of patients who are fully awake gradually increases with increasing stimulation frequency. Notably, when the SCS electrode deviates from the midline, it can easily cause unilateral limb movement and patient discomfort. Such circumstances may require clinicians to reduce the stimulation intensity or remove the stimulating electrode, affecting the patients' outcomes.

## 5 LIMITATIONS

This study is retrospective, and patients served as their own controls. Due to the limitations inherent to the study design, it is difficult to exclude confounding factors that may influence the therapeutic effect, especially to the point of completely distinguishing the therapeutic effect from spontaneous recovery. Collectively, most of our surgical patients had reached a plateau of recovery and had undergone thorough evaluation, which increased the reliability of the study. However, the ideal experimental design is a parallel randomized controlled trial. In fact, the clinical situation of patients with DoC is very complicated, and there are many difficulties in setting up negative controls in such studies and achieving credible results. Subsequent studies should continue to make efforts in this direction, use improved experimental designs, and conduct prospective controlled studies on large samples to strengthen the results.

## 6 CONCLUSION AND PROSPECTS

SCS is one of the most feasible therapeutic schemes to promote awakening in patients with DoC, especially for patients with MCS and patients whose condition is caused by trauma. The current procedures for assessing consciousness and selecting stimulation parameters are mainly based on clinical observation, which will gradually be replaced by evaluation with more objective detection technology in the near future. Based on comprehensive studies of brain networks and the mechanisms of DoC, stimulation paradigms should be explored further in order to improve patients' outcomes. Further, based on auxiliary technologies, individualized parameters for stimulation paradigms will be set according to the characteristics of DoC, thus realizing a closed‐loop stimulation strategy and data mining to improve the effect of neuromodulation therapy.

## 7 AUTHOR CONTRIBUTIONS

Y.Y. and J.Z. designed the study. Y.Y. and Q.H. wrote the manuscript and performed the statistical analysis. X.X., Y.D., and X.C. collected the data. J.H. reviewed the manuscript.

## 9 CONFLICT OF INTEREST

The authors declare that they have no conflicts of interest.

10

## Supporting information


Figure S1

Table S1

## Data Availability

The data that support the findings of this study are available on request from the corresponding author.
