# Supplementary material for: Long‐term functional prognosis and related factors of spinal cord stimulation in patients with disorders of consciousness
Source: CNS Neurosci Ther. 2022 May 20;28(8):1249–58. doi: 10.1111/cns.13870 (PMC9253730; doi:10.1111/cns.13870)
Supplement: Supplementary file 2 — Table S1 [file CNS-28-1249-s002.docx]

Supplementary Table 1. Patient Information

| No. | Hospitalization  number | Gender | Diagnosis | Pathogeny | Duration (months) | Age (years) | Outcome |
| --- | --- | --- | --- | --- | --- | --- | --- |
| 1 | 706313 | M | PVS | stroke | 15 | 38 | dead |
| 2 | 706400 | F | PVS | stroke | 4 | 62 | dead |
| 3 | 701516 | M | PVS | anoxia | 5 | 42 | effective |
| 4 | 701651 | M | PVS | anoxia | 8 | 47 | unchanged |
| 5 | 712252 | M | PVS | stroke | 4 | 50 | dead |
| 6 | 715072 | F | PVS | trauma | 11 | 49 | unchanged |
| 7 | 716620 | F | MCS | trauma | 33 | 30 | effective |
| 8 | 716899 | M | PVS | trauma | 5 | 23 | unchanged |
| 9 | 721259 | M | PVS | trauma | 84 | 47 | unchanged |
| 10 | 731307 | M | PVS | trauma | 7 | 60 | unchanged |
| 11 | 725938 | F | PVS | trauma | 4 | 49 | effective |
| 12 | 734871 | F | MCS | anoxia | 26 | 60 | effective |
| 13 | 740182 | M | PVS | trauma | 4 | 58 | unchanged |
| 14 | 750826 | F | MCS | anoxia | 9 | 62 | effective |
| 15 | 745963 | M | PVS | trauma | 6 | 36 | unchanged |
| 16 | 750521 | F | PVS | trauma | 44 | 24 | effective |
| 17 | 755342 | F | PVS | trauma | 3 | 18 | effective |
| 18 | 759022 | M | PVS | trauma | 12 | 58 | unchanged |
| 19 | 755366 | M | PVS | anoxia | 3 | 53 | unchanged |
| 20 | 759453 | M | PVS | trauma | 16 | 27 | unchanged |
| 21 | 762692 | M | PVS | anoxia | 4 | 61 | unchanged |
| 22 | 623827 | F | PVS | trauma | 77 | 46 | unchanged |
| 23 | 767260 | F | PVS | anoxia | 3 | 25 | unchanged |
| 24 | 779032 | M | PVS | anoxia | 6 | 50 | unchanged |
| 25 | 779035 | M | PVS | anoxia | 11 | 57 | unchanged |
| 26 | 779423 | F | PVS | anoxia | 3 | 28 | unchanged |
| 27 | 789684 | M | MCS | trauma | 8 | 61 | effective |
| 28 | 795633 | M | PVS | anoxia | 5 | 52 | unchanged |
| 29 | 811304 | F | PVS | stroke | 3 | 58 | dead |
| 30 | 809910 | M | PVS | trauma | 7 | 36 | effective |
| 31 | 812783 | F | PVS | stroke | 6 | 11 | effective |
| 32 | 813390 | M | PVS | trauma | 6 | 52 | unchanged |
| 33 | 819179 | F | PVS | anoxia | 3 | 42 | unchanged |
| 34 | 827101 | F | PVS | trauma | 14 | 46 | effective |
| 35 | 827432 | F | PVS | anoxia | 5 | 52 | unchanged |
| 36 | 826705 | F | PVS | anoxia | 3 | 35 | unchanged |
| 37 | 833547 | M | MCS | anoxia | 4 | 42 | effective |
| 38 | 833479 | M | PVS | trauma | 18 | 43 | effective |
| 39 | 833854 | F | MCS | trauma | 7 | 39 | unchanged |
| 40 | 835365 | F | PVS | trauma | 23 | 15 | unchanged |
| 41 | 840173 | M | PVS | stroke | 3 | 52 | unchanged |
| 42 | 849237 | M | PVS | trauma | 4 | 28 | unchanged |
| 43 | 849602 | M | PVS | trauma | 6 | 71 | unchanged |
| 44 | 849771 | M | PVS | anoxia | 3 | 43 | effective |
| 45 | 857645 | M | PVS | anoxia | 8 | 45 | unchanged |
| 46 | 857829 | F | PVS | trauma | 11 | 20 | effective |
| 47 | 857794 | F | MCS | trauma | 4 | 31 | unchanged |
| 48 | 857807 | F | PVS | stroke | 3 | 32 | unchanged |
| 49 | 857158 | M | MCS | trauma | 9 | 34 | effective |
| 50 | 857413 | M | MCS | trauma | 8 | 40 | effective |
| 51 | 864019 | F | MCS | stroke | 3 | 58 | unchanged |
| 52 | 849292 | F | MCS | stroke | 6 | 47 | effective |
| 53 | 864769 | F | MCS | trauma | 12 | 32 | effective |
| 54 | 865081 | M | MCS | trauma | 39 | 22 | effective |
| 55 | 865315 | M | MCS | stroke | 3 | 45 | unchanged |
| 56 | 939584 | F | MCS | trauma | 5 | 17 | effective |
| 57 | 917625 | M | PVS | stroke | 8 | 42 | dead |
| 58 | 939671 | M | PVS | stroke | 3 | 46 | unchanged |
| 59 | 868914 | M | MCS | stroke | 8 | 40 | effective |
| 60 | 868886 | M | PVS | anoxia | 13 | 17 | dead |
| 61 | 868737 | F | MCS | anoxia | 28 | 29 | effective |
| 62 | 939864 | M | MCS | anoxia | 9 | 18 | unchanged |
| 63 | 873234 | M | PVS | anoxia | 4 | 41 | unchanged |
| 64 | 873412 | M | PVS | stroke | 12 | 54 | effective |
| 65 | 833785 | F | PVS | stroke | 18 | 65 | unchanged |
| 66 | 883966 | M | PVS | trauma | 15 | 53 | effective |
| 67 | 869153 | M | PVS | stroke | 7 | 41 | unchanged |
| 68 | 894843 | F | MCS | anoxia | 4 | 27 | effective |
| 69 | 906700 | M | PVS | anoxia | 3 | 52 | unchanged |
| 70 | 892524 | M | PVS | stroke | 3 | 40 | unchanged |
| 71 | 893150 | M | PVS | stroke | 8 | 43 | unchanged |
| 72 | 894499 | M | PVS | anoxia | 5 | 29 | effective |
| 73 | 907388 | M | PVS | stroke | 3 | 42 | unchanged |
| 74 | 907311 | F | MCS | stroke | 5 | 38 | effective |
| 75 | 907124 | M | PVS | stroke | 3 | 52 | unchanged |
| 76 | 892972 | M | MCS | trauma | 3 | 9 | effective |
| 77 | 907310 | F | PVS | stroke | 3 | 54 | unchanged |
| 78 | 894254 | M | MCS | stroke | 6 | 40 | unchanged |
| 79 | 892897 | F | MCS | trauma | 3 | 18 | effective |
| 80 | 910459 | M | PVS | anoxia | 11 | 30 | unchanged |
| 81 | 914322 | M | MCS | stroke | 4 | 61 | unchanged |
| 82 | 910359 | M | MCS | stroke | 4 | 53 | unchanged |
| 83 | 907318 | M | MCS | anoxia | 6 | 30 | unchanged |
| 84 | 914667 | M | MCS | stroke | 3 | 51 | effective |
| 85 | 914153 | F | PVS | stroke | 3 | 65 | dead |
| 86 | 914633 | M | PVS | trauma | 4 | 44 | unchanged |
| 87 | 914737 | M | PVS | anoxia | 21 | 11 | effective |
| 88 | 925076 | M | PVS | anoxia | 6 | 24 | unchanged |
| 89 | 925472 | F | PVS | stroke | 7 | 39 | unchanged |
| 90 | 927893 | M | MCS | stroke | 9 | 54 | unchanged |
| 91 | 927823 | M | MCS | trauma | 5 | 44 | unchanged |
| 92 | 927886 | M | PVS | stroke | 5 | 41 | effective |
| 93 | 929513 | F | PVS | trauma | 4 | 40 | unchanged |
| 94 | 925155 | M | MCS | trauma | 4 | 26 | effective |
| 95 | 929709 | M | PVS | anoxia | 3 | 42 | unchanged |
| 96 | 929639 | F | PVS | stroke | 12 | 29 | unchanged |
| 97 | 929864 | M | PVS | trauma | 8 | 33 | unchanged |
| 98 | 929129 | M | PVS | anoxia | 3 | 32 | unchanged |
| 99 | 940198 | M | PVS | trauma | 8 | 58 | unchanged |
| 100 | 929792 | M | PVS | trauma | 3 | 47 | unchanged |
| 101 | 940213 | M | PVS | stroke | 3 | 49 | unchanged |
| 102 | 938974 | M | PVS | anoxia | 3 | 53 | unchanged |
| 103 | 940836 | M | PVS | trauma | 24 | 18 | unchanged |
| 104 | 929933 | F | PVS | anoxia | 4 | 30 | unchanged |
| 105 | 940684 | M | PVS | stroke | 8 | 55 | unchanged |
| 106 | 939296 | F | PVS | anoxia | 41 | 35 | unchanged |
| 107 | 939155 | M | PVS | trauma | 4 | 57 | unchanged |
| 108 | 939430 | M | PVS | stroke | 5 | 44 | unchanged |
| 109 | 959599 | M | PVS | stroke | 3 | 42 | unchanged |
| 110 | 959518 | M | MCS | trauma | 8 | 33 | effective |
